# Cost-Effectiveness of an Intervention to Reduce Emergency Re-Admissions to Hospital among Older Patients

**DOI:** 10.1371/journal.pone.0007455

**Published:** 2009-10-14

**Authors:** Nicholas Graves, Mary Courtney, Helen Edwards, Anne Chang, Anthony Parker, Kathleen Finlayson

**Affiliations:** 1 School of Public Health, Institute of Health and Biomedical Innovation, Queensland University of Technology, Brisbane, Australia; 2 Faculty of Health, Queensland University of Technology, Brisbane, Australia; 3 School of Nursing and Midwifery, Institute of Health and Biomedical Innovation, Queensland University of Technology, Brisbane, Australia; 4 Queensland Centre for Evidence Based Nursing & Midwifery, Mater Health Services, and School of Nursing, Institute of Health and Biomedical Innovation, Queensland University of Technology, Brisbane, Australia; 5 School of Human Movement Studies, Institute of Health and Biomedical Innovation, Queensland University of Technology, Brisbane, Australia; Canadian Agency for Drugs and Technologies in Health, Canada

## Abstract

**Background:**

The objective is to estimate the cost-effectiveness of an intervention that reduces hospital re-admission among older people at high risk. A cost-effectiveness model to estimate the costs and health benefits of the intervention was implemented.

**Methodology/Principal Findings:**

The model used data from a randomised controlled trial conducted in an Australian tertiary metropolitan hospital. Participants were acute medical admissions aged >65 years with at least one risk factor for re-admission: multiple comorbidities, impaired functionality, aged >75 years, recent multiple admissions, poor social support, history of depression. The intervention was a comprehensive nursing and physiotherapy assessment and an individually tailored program of exercise strategies and nurse home visits with telephone follow-up; commencing in hospital and continuing following discharge for 24 weeks. The change to cost outcomes, including the costs of implementing the intervention and all subsequent use of health care services, and, the change to health benefits, represented by quality adjusted life years, were estimated for the intervention as compared to existing practice. The mean change to total costs and quality adjusted life years for an average individual over 24 weeks participating in the intervention were: cost savings of $333 (95% Bayesian credible interval $ -1,932∶1,282) and 0.118 extra quality adjusted life years (95% Bayesian credible interval 0.1∶0.136). The mean net-monetary-benefit per individual for the intervention group compared to the usual care condition was $7,907 (95% Bayesian credible interval $5,959∶$9,995) for the 24 week period.

**Conclusions/Significance:**

The estimation model that describes this intervention predicts cost savings and improved health outcomes. A decision to remain with existing practices causes unnecessary costs and reduced health. Decision makers should consider adopting this program for elderly hospitalised patients.

## Introduction

Hospital bed days are a valuable economic commodity. In 2004/05 Australian policy makers allocated expenditures of $14,470 (AUD) million to supply 14,391 hospital bed days, yet waiting lists continue to grow. In 2004/05 ninety percent of patients were admitted for elective surgery within 217 days compared to 197 days in 2002/03 [1]. The adoption of novel health programmes that reduce risk of hospital re-admission are worth considering on economic grounds. Programmes will incur a positive cost to implement but cost savings may arise after adoption and extra health benefits may result from reduced morbidity and mortality risk. The overall change to costs and benefits need to be weighed up, and for this purpose health economists have developed evaluation methods.

The basics of economic evaluation in healthcare are described by Donaldson et al. [2] and Drummond et al. [3] provide a useful text book. The concept of opportunity cost is the mainstay. This shows that using scarce resources for one programme incurs a cost, because an opportunity to pursue some other beneficial programme is lost. An efficient outcome is when resources are allocated across programmes such that opportunity cost is minimised, which is analogous to benefit being maximised. Providing healthcare decision makers with information about the changes to costs and benefits from adopting different health programmes can improve efficiency.

A primary method for economic evaluation is cost-benefit analysis that summarises in monetary values the gains and losses from adopting a novel health programme. If costs are found to be less than the benefits then the programme is desirable, and programmes with the highest net benefits are most desirable [4]. The advantage of this approach is that all manner of different outcomes, some non-health related, can be included in an evaluation. The findings should represent an aggregation of the values of all members of society and provide information about opportunity cost. There are difficult challenges however for the analyst who attempts to find monetary valuations for costs and benefits that change with the adoption of a new health programme. Individuals find it difficult to reveal accurately how they value health improvement in monetary terms [5], and, those with more money may give different responses, skewing the allocation of resources toward the wealthy [6]. A number of cost-benefit analyses of health care programmes have been published in the medical and economics literature and these have been reviewed [7], [8].

Another method for economic evaluation is cost-effectiveness analysis. In this case the analyst does not seek a monetary valuation of health benefit but counts benefit in natural units of outcome such as years of life saved, pain free days or quantifiable improvements in symptoms. This method is easier to apply and disseminate. A disadvantage is that the connection with opportunity costs is severed as individual valuations are not included. It has been suggested that cost-effectiveness analyses can only address questions about whether the same level of health output can be achieved at lower cost [2]. Cost-effectiveness analysis may be less useful than cost-benefit analysis because it cannot directly address questions about how resources are allocated between programmes.

A special type of cost-effectiveness analysis is cost-utility analysis. The analyst measures health benefit by quality adjusted life years (QALYs). These are extra years of life gained from a novel programme weighted by a preference based value between zero (dead) and one (good health) [9]. Five years of extra life valued at 0.5 per year by individuals will equal 2.5 QALYs. Decision makers may be attracted to programmes that provide a cheaper marginal QALY. An advantage of cost-utility analysis is that the weighting score, that updates the number of QALYs gained, represents individual preferences or utilities [10]. This allows decision makers to pursue the objective of maximising health gain (QALYs) from a fixed pot of health resources. This approach to decision making in healthcare is called extra-welfarism and has been embraced by the institutional regulators of publically funded health care systems in the UK [11] and Australia [12], as well as in other settings. The decision rule can be summarised in simple terms. If the change in health costs (ΔC) divided by the change in QALYs (ΔE) is less than some threshold value that health decision makers are willing to pay for the marginal QALY (γ), then the programme is cost-effective and should be adopted:
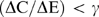



It is useful to consider uncertainty in health decision making [13] and ratios have awkward statistical properties. This is resolved by rearranging the same information to calculate a net-monetary-benefit statistic [14]:
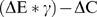



This provides a linear outcome, rather than a ratio, and facilitates quantitative analyses of data that are easier to interpret for decision making [15].

Cost-utility analyses do not provide the same information that would arise from a cost-benefit analysis, for which the analyst elicits individual valuations of outcomes. Instead, the value per QALY (γ) arises from a judgement made by government or some quasi-government agency who aim to maximize health from scarce resources [16]. Health economists debate the merits of the different approaches to economic evaluation [17]. If decision makers want the largest public benefit then cost-benefit analysis is preferred as opportunity costs are made explicit; but valuing individual preferences for health in money terms is problematic [5], [6]. If decision makers aim to maximise the amount of health gain (i.e., QALYs) from a fixed pot of health resources then the cost-utility analysis approach may be useful [18]. Birch and Gafni [19] make an important point that cost-utility analyses tend to focus on individual programmes rather than opportunity cost, and so the important issue of affordability is neglected. Instead of thinking about affordability, decision makers working with cost-utility information use the maximum willingness to pay for a marginal QALY (γ) as a decision rule. This value varies in practice, often to accommodate other important objectives such as equity and fairness [11]. Some argue it is arbitrary and that extra-welfarism is unlikely to lead to an optimal allocation of healthcare resources [20]. Solutions have been proposed that utilise a mathematical programming method [21] and this method has been applied to a real resource allocation problem [20]. If cost-utility analysis is used to guide decisions then the impact on the overall health budget should be described and the issue of affordability made explicit [2], [22].

The aim of this paper is to describe a cost-utility analysis of a decision to adopt a novel health programme that has been shown to reduce re-admissions to hospital among an elderly and high risk group. The data arise from a randomised controlled trial of the intervention conducted in an Australian setting [23]. The analyses are motivated by an extra-welfarist goal of maximising health benefits from a health budget. The research question is whether scarce healthcare resources should be allocated to provide this intervention, or whether remaining with existing practice is a better decision. The implications of using cost-utility analysis to address this policy question are discussed. Older adults have higher rates of hospital admission and re-admission than the general population in Australia [24] the UK [25] and the US [26]. Preventing emergency re-admissions among older patients is one way to ease pressure on beds in acute hospitals and save costs. A recent randomised controlled trial conducted in an Australian setting showed an intervention was effective at preventing emergency re-admission to hospital among older people with known risk factors for re-admission [23]. The economic outcomes of adopting this intervention have not been described and may provide useful information for health decision makers.

## Methods

The aim of the primary trial was to measure the effect of an intervention targeting patients at high risk of hospital re-admission on health service utilisation, health-related quality of life, general health, psycho-social outcomes and functional ability. Participants were recruited into the trial within 72 hours of hospital admission and randomised to intervention or control group. The control group (n = 64) received the routine discharge planning and rehabilitation advice, this is defined as usual care. The intervention group (n = 58) received additional intervention described in detail by Courtney et al. [23]. Within 72 hours of admission a nurse and a physiotherapist made a comprehensive patient assessment and together wrote an individual post-discharge care plan. This included telephone follow up by a nurse and an exercise programme of muscle stretching, balance training, walking for endurance, and muscle strengthening using resistance exercises. During the inpatient stay the nurse visited patients every day to address concerns, facilitate the exercise program, and oversee discharge planning. Written guidelines were provided on post-discharge management, including diagrams and specific instructions for their exercise program. Within 48 hours of discharge the nurse visited the patient in their home to assess their progress. Extra home visits were provided if required. One telephone call was provided each week for four weeks, followed by monthly calls until 24 weeks. The nurse was also available for contact between 9 a.m. and 5 p.m. weekdays. Issues that might impede adherence and progress were addressed during the telephone follow-up. Data that describe patient outcomes were collected at baseline and then at 4, 12, and 24 weeks after discharge.

The mean age of the groups was 78.1 years for intervention and 79.4 for controls; 36% and 40% were male, mean lengths of stay were 4.6 and 4.7 days and frequencies of co-morbidities and risk factors for re-admission were similar. Baseline characteristics are reported by Courtney et al. [23]. There were significantly fewer emergency hospital re-admissions among the intervention group (22% of intervention group, 47% of control group, p = .007) and significant fewer emergency GP visits (25% of intervention group, 67% of control group, p<.001). The intervention group showed significantly greater improvements in quality of life over the control group as measured by the SF-12v2 health survey.

**Figure 1:**
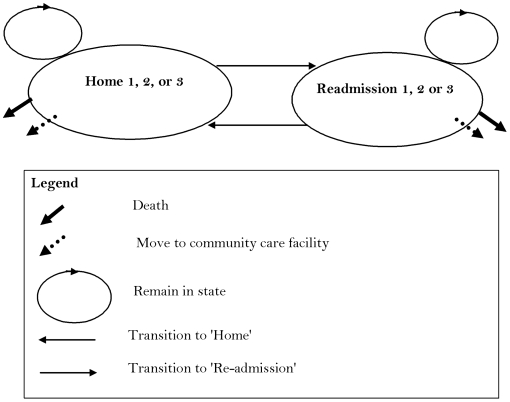
State transition Markov model.

The perspective for the cost-effectiveness model was the health care provider in the Australian setting and costs to patients and informal were not measured, nor were productivity changes. The patient level data collected by Courtney et al. [23] were used alongside other routinely available data to inform a decision analytic cost-effectiveness model. The modelling process compared a decision to adopt the novel intervention as compared to a decision to remain with a usual care alternative (i.e. the control group in the trial). The progress of all patients after discharge from a primary hospital admission was described using a Markov state-transition process [27] shown in Figure 1. The advantage of a Markov model is that it can quantify a decision problem that involves risk over time, when the timing of events is important and when important events may happen more than once [28]. As time moves forward patients face some probability of remaining at home, transitioning to a community care facility, being re-admitted to hospital or dying. Transition to a community care facility and death are one way moves whereas patients can cycle in and out of hospital up to three times during the 24 weeks of the study. The model updates every day for a period of 24 weeks after discharge. All transition probabilities are labelled in Figure 1. For each day in the model costs and health benefits (QALYs) accumulate based on the patient level data collected by Courtney et al. [23]. The cost and QALY outcomes are summed at the end of the 24 week trial and a comparison drawn between the patients who received the intervention and those who received usual care. The difference in costs (ΔC) and QALYs (ΔE) are combined with valuations of the willingness to pay for a marginal QALY (γ) to estimate net-monetary-benefits for a decision to adopt the intervention (i.e., (ΔE * γ) - ΔC)). The maximum willingness to pay for a QALY was assumed to be $64,000 for the Australian setting [29].

All cost outcomes are reported in 2008 Australian dollars. The costs of the intervention were incurred during the primary hospital admission and then after discharge. The primary admission costs include the time of the nurse and physiotherapist who worked with each patient and prepared an individual exercise plan for them to take home. The post-discharge costs include a single home visit by a programme nurse and ten follow up calls of 20–30 minutes each over 24 weeks. Patients were also given an exercise stretchy band and pedometer to assist with the prescribed physical activities. After discharge from the primary admission the costs of GP visits, hospital emergency department visits or utilisation of ‘other’ health care services such as physiotherapy, home help and community nursing were included. The number of days spent in hospital following a re-admission to hospital or admission to a community nursing facility was included, and the relevant cost per day applied. Values for all cost parameters are shown in Table 1.

**Table 1:** Data Used to Estimate Costs of Delivering the Intervention per Patient, all costs in 2008 ▒AUD.

Cost Item	Data Used	Source
Assessment by Physiotherapist	Consultation between 80 and 150 minutes	Notes kept by intervention physiotherapist
Assessment by Nurse	Consultation between 30 and 60 minutes	Notes kept by intervention nurse
Daily Visits by Nurse	Consultation between 10 and 30 minutes	Notes kept by intervention nurse
Length of stay for primary admission	Mean = 4.66 days, St Dev = 2.77 days	Data collected by Courtney et al. [23]
One off home visit	Travel and visit time between 100 and 150 minutes	Notes kept by intervention nurse
Ten follow up calls over 6 months	Duration of call between 20 and 30 minutes	Notes kept by intervention nurse
▒10 stretchy band	▒10	Data collected by Courtney et al. [23]
▒10 pedometer	▒10	Data collected by Courtney et al. [23]
Hourly cost Physiotherapist	▒52.3	Mater Health Services, salary schedule
Hourly cost senior Nurse (HEWA 7)	▒50	Mater Health Services, salary schedule
GP, ED and other health care service per visit	▒50 to ▒90	Medical Benefits Schedule [44]
Value of bed day in hospital	▒611 to ▒1008	Australian Hospital Statistics [45]
Value of bed day in community care facility	▒108	Data provided by Economics and Health Services Group (AIHW), and [46]

The SF-12 health survey outcomes collected at baseline, 4, 12 and 24 weeks were mapped onto EQ-5D utility values using an algorithm developed by Gray et al. [30]. This estimates a utility score between zero and 1 to describe the value of the health state of patients. These utility scores were profiled over the 24 weeks of the study to estimate the number of quality adjusted life years (QALYs). The risk of death observed among the sample was used to describe the risks of death for all model participants.

Uncertainties in the data were propagated forward to the results by fitting probability distributions to each parameter. Beta distributions were fitted to all transition probabilities and gamma distributions to all cost parameters. Uniform distributions were fitted to parameters where only two data points were available that describe a high and low value. One thousand random re-samples were taken from all distributions. This gave rise to 1000 estimates of the change to total cost (ΔC), change to total QALYs (ΔE) and net-monetary-benefit. The probability the intervention was cost-effective as compared to the usual care alternative was estimated by counting the number of times the net-monetary-benefit statistic was positive over the total number of re-samples [31]. These results were plotted as cost-effectiveness acceptability curves that show the probability that adopting the intervention is a cost-effective decision, given uncertainties in model parameters. Cost-effectiveness acceptability curves describe a wide range of decision maker's willingness to pay for QALYs (γ). Detailed information about the use and interpretation of cost-effectiveness acceptability curves is available [31], [32]. The process captures the variance in the data collected from trial participants and accounts for any correlations between parameters. The method used to fit beta, gamma and uniform distributions to the data and the method used estimate daily probabilities is described in the Appendix S1.

## Results

The mean cost of delivering the intervention was $547 (95% Bayesian credible interval $470∶$626) per individual. The mean daily probabilities of re-admission to hospital for the intervention and usual care conditions during the 24 weeks after primary discharge are shown in Table 2. Between week 1 and week 4 the usual care condition faced a higher daily probability of a first re-admission to hospital. There were zero second or third re-admissions. Between week 5 and 12 the usual care group faced a marginally lower daily probability of a first re-admission, but a higher probability of a second re-admission. Between week 13 and 24 the usual care group faced a higher probability of a first and third re-admission, but not for the second re-admission. For the entire 24 week period the daily probabilities of transitioning into a community care facility were 0.00039% for the usual care group and 0.00016% for the intervention group. The use of non-hospital based health services were higher for the usual care condition across all time periods and types of services, with the exception of ‘Visits to Emergency Department’ during weeks 1 to 4 after discharge. During this time period the intervention group used more services than the usual care condition. These results are shown in Table 3.

**Table 2:** Daily probabilities (95% Bayesian credible interval in brackets) of re-admission to hospital for 24 weeks following primary discharge for the intervention and usual care conditions.

	First re-admission (%)	Second re-admission (%)	Third re-admission (%)
Week 1 to 4 - Usual care	0.405 (0.395∶0.415)	Zero re-admissions	Zero re-admissions
Week 1 to 4 - Intervention	0.313 (0.303∶0.323)	Zero re-admissions	Zero re-admissions
Week 5 to 12 - Usual care	0.216 (0.211∶0.220)	0.281 (0.271∶0.290)	Zero re-admissions
Week 5 to 12 - Intervention	0.259 (0.254∶0.264)	Zero re-admissions	Zero re-admissions
Week 13 to 24 - Usual care	0.131 (0.128∶0.134)	0.077 (0.075∶0.080)	0.040 (0.038∶0.041)
Week 13 to 24 - Intervention	0.028 (0.026∶0.029)	0.188 (0.182∶0.194)	Zero re-admissions

**Table 3:** Mean number (95% Bayesian credible interval in brackets) of consultations with non-hospital services for 24 weeks following primary discharge for the intervention and usual care conditions.

	GP consultations	Visits to emergency department	All other health contacts #
Week 1 to 4 - Usual care	0.45 (0.26∶0.70)	0.12 (0.06∶0.21)	1.12 (0.34∶2.41)
Week 1 to 4 - Intervention	0.12 (0.04∶0.23)	0.18 (0.07∶0.31)	0.07 (0.01∶0.17)
Week 5 to 12 - Usual care	0.25 (0.12∶0.43)	0.17 (0.08∶0.28)	0.22 (0.02∶0.68)
Week 5 to 12 - Intervention	0.05 (0.01∶0.12)	0.03 (0.00∶0.09)	0.11 (0.00∶0.38)
Week 13 to 24 - Usual care	0.64 (0.41∶0.91)	0.12 (0.05∶0.23)	0.28 (0.03∶0.74)
Week 13 to 24 - Intervention	0.07 (0.02∶0.15)	0.07 (0.02∶0.15)	0.12 (0.01∶0.41)

# contact with the outpatients department, visiting a chemist, physiotherapy, community nursing and home help service.

**Figure 2:**
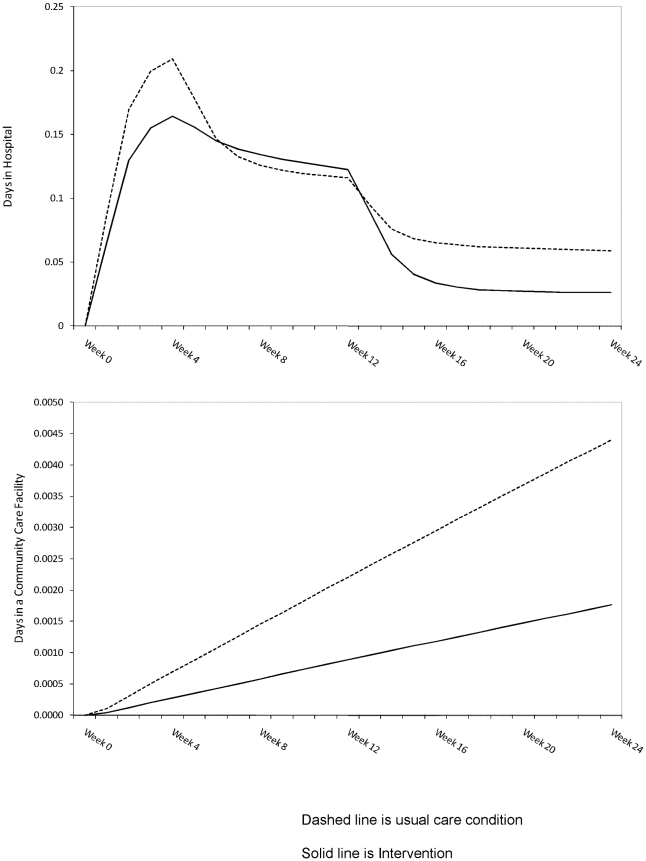
Number of days spent in an acute hospital and community care facility.

The number of days the average individual spent in a hospital bed and a community care facility for the intervention and usual care conditions are shown in Figure 2 for the 24 weeks of data collection; the mean difference in the number of bed days used for this time period is 0.48 days. For the average individual, participating in the intervention for 24 weeks reduced costs by $333 (95% Bayesian credible interval $-1,932∶1,282) and increased QALYs by 0.118 (95% Bayesian credible interval 0.1∶0.136). The mean net-monetary-benefit per individual for the intervention group compared to the usual care condition was $7,907 (95% Bayesian credible interval $5,959∶$9,995) for the 24 week period.

**Figure 3:**
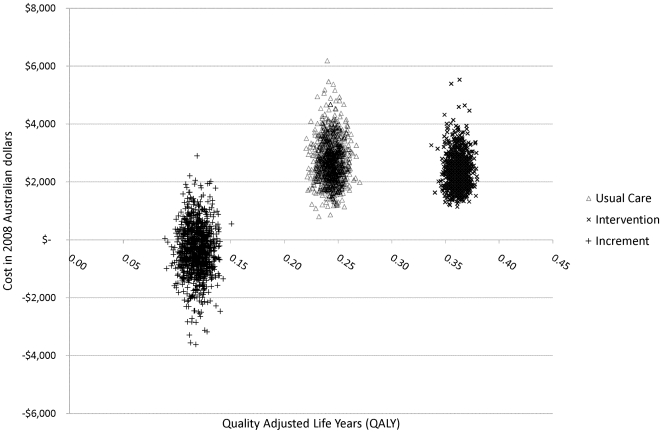
Joint distribution of cost and QALY outcomes.

**Figure 4:**
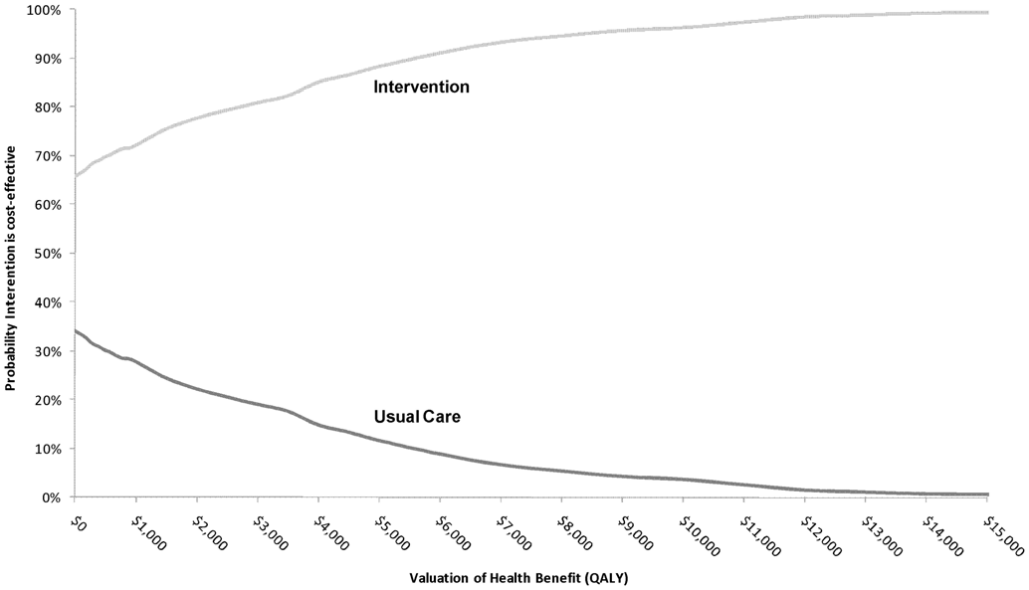
Cost-effectiveness acceptability curves.

Uncertainties arising from the parameters used in the modelling are shown in Figure 3. This reveals clear improvement among health outcomes for the Intervention group with all re-samples for QALY values higher for the usual care condition. For the overall change to cost outcome, 640 out of the 1000 re-samples took a negative value. The interpretation of these findings is that there is a 100% probability the intervention delivers higher health benefits and a 64% chance the intervention saves costs. These are summarised in the cost-effectiveness acceptability curve included in Figure 4. The intervention always has the highest change of being the cost-effective decision, regardless of the value chosen for health benefits (QALYs).

## Discussion

The results show that from a health service perspective net-monetary benefits are almost $8,000 per individual who is offered the intervention programme. We expect the opportunity cost to health services from adopting this intervention to be negligible because cost savings are likely to compensate the positive costs of implementing the programme. In this case the impact on the overall health budget should be neutral and costs may even be saved; the issue of affordability is not paramount. Gains in health benefits are enjoyed by those offered the intervention. The results are robust to the uncertainty among the model parameters used. The lower Bayesian credible interval for monetary-net-benefits takes a positive value of $5,959 per individual. Based on the data available it appears that intervening with a nursing and physiotherapy assessment and then following up after discharge is a sensible decision on economic grounds.

The policy implications of this are substantial. In 2005–06 there were 2,594,755 patient separations from all Australian hospitals for individuals aged over 65 years (Table 7.6 in Australian Hospital Statistics [33]). Unpublished data collected for the original trial [23] showed that close to 50% of the >65 years population discharged from hospital would meet the eligibility criteria for the trial (Personal communication, Mary Courtney, August 6, 2009). This information suggests more than one million individuals could derive net benefits from receiving this intervention annually.

Other studies have evaluated models of discharge planning and follow-up care programs for older patients [34]–[38], yet few consider whether the intervention is cost-effective. There appears to be a shortage of cost-effectiveness data in this area. The studies most frequently reporting cost-effectiveness outcomes are evaluations of transitional care programs for patients with chronic heart failure. These programs aim to prevent hospital re-admissions and morbidity in this population, though the program components and outcomes vary considerably. One report of a program utilizing regular community nurse home visits for six months following hospital discharge did not result in significantly decreased re-admission rates and no economic benefit [39]. Another intervention with nurse-led telephone contacts found decreased hospitalization costs, but a slight increase in outpatient department visits [40]. Two studies reported positive economic outcomes; one with a program of regular Day Hospital follow-up visits after discharge in comparison to usual outpatient community care [41]; the other a more intensive program combining hospital specialist care and discharge planning, outpatient follow-up visits, nurse phone follow-up and home GP visits [42]. The Day Hospital follow-up care resulted in decreased re-admissions, morbidity and mortality; and incremental cost effectiveness analysis found $1,068 savings for each quality-adjusted life year gained [41]. The program combining hospital, outpatient, telephone follow-up and home visits is similar in scope to the study reported here, and also found decreased re-admissions and morbidity, with mean cost savings of €982 per patient [42]. Only one study was found that evaluated an intervention for patients with mixed medical diagnoses, and this study included all medical patients regardless of age or presence of risk factors for re-admission. The study of a nurse-led, home-based, case management intervention for 24 weeks after discharge found no differences in outcomes, including total costs [43].

There are caveats to this research. Not all costs were considered, and the perspective of this analysis was the health care provider. Costs to informal carers such as time spent by friends and family members looking after participants were not included, nor were productivity changes among participants. It is possible that the intervention group incurred higher informal carer costs because they spent less time in hospital or community care facilities. It is also likely that with fewer re-admissions and better health outcomes there were productivity gains among the intervention group. These values are not described by the data used in this modelling study. We only followed patients for 24 weeks after discharge and the benefits of the intervention may reduce after this time. The question of whether data from a relatively modest sample are sufficient to generalise to other settings is important. Answers would emerge from repeating the study using larger samples. Any biases from the primary trial would however have to be substantial for the decision to change from simply recruiting a larger sample.

It is valuable for decision makers to find an intervention that dominates their existing practice by both measures of cost and effectiveness. Based on the current data this intervention represents a ‘win-win’ for policy makers. A decision to remain with existing practice implies higher cost outcomes and worse health outcomes and this should not sit well with those who manage health services. The relatively low cost intervention appears to save costs and improve health outcomes. Patients are less likely to be re-admitted to hospital or a community care facility and this is a major source of cost saving. Uncertainty in these findings could be reduced by repeating the intervention trial using a larger sample of individuals. Decision makers in the Australian setting should consider the economic evidence for adopting this programme presented here, alongside other factors relevant to the adoption decision. Others may be interested in evaluating this decision in other settings as the extent to which these findings can be generalised is unknown.

## Supporting Information

Appendix S1 (0.08 MB RTF)

## References

[1] Australian Institute of Health & Welfare (2006). Australian Hospital Statistics 2004–05..

[2] Donaldson C, Currie G, Mitton C (2002). Cost effectiveness analysis in health care: contradictions.. BMJ.

[3] Drummond MF, Sculpher MJ, Torrance GW, O'Brien BJ, Stoddart GL (2005). Methods for the Economic Evaluation of Health Care Programmes, third edition..

[4] Layard RN, Glaister S, Layard R, Glaister S (1994). Introduction.. Cost-Benefit Analysis.

[5] Cookson R (2003). Willingness to pay methods in health care: a sceptical view.. Health Economics.

[6] Coast J (2004). Is economic evaluation in touch with society's health values?. BMJ.

[7] Ryan M, Scott DA, Reeves C, Bate A, van Teijlingen ER (2001). Eliciting public preferences for health care: a systematic review of techniques.. Health Technol Assess.

[8] Sampietro-Colom L, Phillips VL, Hutchinson AB (2004). Eliciting women's preferences in health care: a review of the literature.. Int J Technol Assess Health Care.

[9] Brazier J, Deverill M, Green C, Harper R, Booth A (1999). A review of the use of health status measures in economic evaluation.. Health Technol Assess.

[10] Torrance GW (1986). Measurement of health state utilities for economic appraisal: a review.. J Health Econ.

[11] Rawlins MD, Culyer AJ (2004). National Institute for Clinical Excellence and its value judgments.. BMJ.

[12] Jackson TJ (2007). Health technology assessment in Australia: challenges ahead.. MJA.

[13] Briggs AH, O'Brien BJ (2001). The death of cost-minimization analysis?. Health Econ.

[14] Hoch JS, Briggs AH, Willan AR (2002). Something old, something new, something borrowed, something blue: a framework for the marriage of health econometrics and cost-effectiveness analysis.. Health Economics.

[15] Claxton K, Sculpher M, McCabe C, Briggs A, Akehurst R (2005). Probabilistic sensitivity analysis for NICE technology assessment: not an optional extra.. Health Econ.

[16] Sugden R, Williams A (1978). The principles of practical cost-benefit analysis..

[17] Krupnick AJ (2004). Valuing health Outcomes: Policy Choices and Technical Issues..

[18] Wagstaff A (1991). QALYs and the equity-efficiency trade-off.. J Health Econ.

[19] Birch S, Gafni A (1992). Cost effectiveness/utility analyses. Do current decision rules lead us to where we want to be?. J Health Econ.

[20] Epstein DM, Chalabi Z, Claxton K, Sculpher M (2007). Efficiency, equity, and budgetary policies: informing decisions using mathematical programming.. Med Decis Making.

[21] Stinnett AA, Paltiel AD (1996). Mathematical programming for the efficient allocation of health care resources.. J Health Econ.

[22] Trueman P, Drummond M, Hutton J (2001). Developing Guidance for Budget Impact Analysis.. Pharmacoeconomics.

[23] Courtney M, Edwards H, Chang A, Parker A, Finlayson K (2009). Fewer emergency re-admissions and better quality of life for older adults at risk of hospital re-admission: a randomized controlled trial to determine the effectiveness of a 24-week exercise and telephone follow-up program.. J Am Geriatr Soc.

[24] Karmel R, Lloyd J, Hales C (2007). Older Australians in Hospital. Bulletin no 53 Cat no AUS 92..

[25] National Health Service (2006). HES online. Hospital Episode Statistics..

[26] Elixhauser A, Yu K, Steiner C, Bierman AS (2000). Hospitalisation in the United States, 1997. Rockville (MD): Agency for Healthcare Research and Quality..

[27] Briggs A, Sculpher M (1998). An Introduction to Markov Modelling for Economic Evaluation.. Pharmacoeconomics.

[28] Sonnenberg FA, Beck JR (1993). Markov models in medical decision making: a practical guide.. Med Decis Making.

[29] Shiroiwa T, Sung YK, Fukuda T, Lang HC, Bae SC (2009). International survey on willingness-to-pay (WTP) for one additional QALY gained: what is the threshold of cost effectiveness?. Health Econ.

[30] Gray AM, Rivero-Arias O, Clarke PM (2006). Estimating the Association between SF-12 Responses and EQ-5D Utility Values by Response Mapping.. Medical Decision Making.

[31] Fenwick E (2004). Cost-effectiveness acceptability curves - facts, fallacies and frequently asked questions.. Health Economics.

[32] Fenwick E, Claxton K, Sculpher M (2001). Representing uncertainty: The role of cost-effectiveness acceptability curves.. Health Economics.

[33] Australian Institute of Health & Welfare (2007). Australian Hospital Statistics 2005–06..

[34] Mistiaen P, Poot E (2006). Telephone follow-up, initiated by a hospital-based health professional, for postdischarge problems in patients discharged from hospital to home.. Cochrane Database of Systematic Reviews.

[35] Naylor MD, Brooten DA, Campbell RL, Maislin G, McCauley KM (2004). Transitional care of older adults hospitalized with heart failure: A randomized, controlled trial.. Journal of the American Geriatrics Society.

[36] Parker SG (2005). Do current discharge arrangements from inpatient hospital care for the elderly reduce re-admission rates, the length of inpatient stay or mortality, or improve health status?.

[37] Phillips CO, Wright SM, Kern DE, Singa RM, Shepperd S (2004). Comprehensive discharge planning with postdischarge support for older patients with congestive heart failure. A meta-analysis.. Journal of the American Medical Association.

[38] Shepperd S, Parkes J, McClaren J, Phillips C (2004). Discharge planning from hospital to home.. Cochrane Database of Systematic Reviews CD000313.

[39] Kwok T, Lee J, Woo J, Lee D, Griffith S (2008). A randomized controlled trial of a community nurse-supported hospital discharge programme in older patients with chronic heart failure.. Journal of Clinical Nursing.

[40] Ho Y, Hsu T, Chen C, Lee C, Lin Y (2007). Improved cost-effectiveness for management of chronic heart failure by combined home-based intervention with clinical nursing specialists.. Journal Of The Formosan Medical Association.

[41] Capomolla S, Febo O, Ceresa M, Caporotondi A, Guazzott G (2002). Cost/utility ratio in chronic heart failure: comparison between heart failure management program delivered by day-hospital and usual care Journal of the American College of Cardiology.

[42] Del Sindaco D, Pulignano G, Minardi G, Apostoli A, Guerrieri L (2007). Two-year outcome of a prospective, controlled study of a disease management programme for elderly patients with heart failure.. Journal Of Cardiovascular Medicine.

[43] Latoura C, Bosmans J, van Tulderb M, de Vosd R, Huysee F (2007). Cost-effectiveness of a nurse-led case management intervention in general medical outpatients compared with usual care: An economic evaluation alongside a randomized controlled trial Journal of Psychosomatic Research.

[44] Department of Health & Ageing (2007). Complete Medicare Benefits Schedule. Australian Government, Department of Health and Ageing..

[45] Australian Institute of Health & Welfare (2006). Australian Hospital Statistics 2004–2005..

[46] Australian Institute of Health & Welfare (AIHW) (2004). Residential Aged Care in Australia 2002–03: A Statistical Overview (ISBN-13 978 1 74024 384 1)..

