# Supplementary material for: Cost-Effectiveness of an Intervention to Reduce Emergency Re-Admissions to Hospital among Older Patients
Source: PLoS One. 2009 Oct 14;4(10):e7455. doi: 10.1371/journal.pone.0007455 (PMC2759083; doi:10.1371/journal.pone.0007455)
Supplement: Appendix S1 — (0.08 MB RTF) [file pone.0007455.s001.rtf]

Appendix S1
Beta Distribution. Use: Prior for proportions/probabilities (takes values between 0 and 1). Notation: Beta(,),mean=/(+). Note interpretation =prior number of successes, =prior number of failures for Binomial probability parameter. 

Gamma Distribution. Use: Likelihood for positive continuous variables, especially those that describe resource use such as length of stay of cost outcomes. Often used as a prior for a Normal precision parameter, and prior for Beta parameters. Notation: Gamma(,), mean=/, variance=/2, shape parameter , scale parameter, . Setting =1 gives the Exponential() distribution. 

Uniform Distribution. Use: Prior for parameters that can only take values on a specific interval (a,b). Often used for the standard deviation, and for proportion parameters (on interval (0,1)). Notation: Unif(a,b), mean = (a+b)/2, variance = (b-a)2/12.

Daily probability of an event was: 1-exponent(daily rate*-1), where, daily rate = -log*(1-resample from beta distribution)/time in study.
